# Engineered Metal-Organic Frameworks (MOFs)-electrospun nanofibers for wound healing: a review

**DOI:** 10.1186/s13036-026-00650-z

**Published:** 2026-03-23

**Authors:** Mohamed A. Abdelkhalek, Mohamed M. Abdelaal, Sara A. Abdel Gaber

**Affiliations:** 1 Institute of Nanoscience and Nanotechnology, Kafrelsheikh University, Kafrelsheikh, 33516 Egypt; 2 Cardiothoracic Surgery Department, Medicine Faculty, Kafrelsheikh University, Kafrelsheikh, 33516 Egypt; 3 Nanomedicine Department, Institute of Nanoscience and Nanotechnology, Kafrelsheikh University, Kafrelsheikh, 33516 Egypt

**Keywords:** Metal-Organic Frameworks (MOFs), Electrospun nanofibers, Wound healing, Multifunctional biomaterial, Machine learning

## Abstract

Metal-Organic Framework (MOF)/nanofiber composites represent a significant evolution in advanced wound care, offering a multi-functional platform for controlled therapeutic delivery and accelerated tissue regeneration. The superior performance of these systems is frequently reported with nearly 100% wound closure within 14 days and a broad-spectrum antimicrobial efficacy, yet the clinical translation remains contingent upon addressing technical and toxicological constraints. On the top of those constraints are the lack of long term stability data and precise dosing protocols in complex wound environments. This review provides a critical evaluation of these composites, analyzing established nanofibers impeded with zinc, copper, silver, zirconium alongside emerging iron -based MOFs that provide superior biocompatibility and enzymatic ‘chemodynamic’ activity, and cobalt systems which offer unique hypoxia-mimicking properties for angiogenesis. In this review the stability-toxicity paradox inherent in ion-releasing MOFs/nanofiber composites is emphasized. We further examine the structural hurdles of fabrication, specifically the rheological disruptions and interfacial mismatches that occur during electrospinning. Furthermore, the role of Machine Learning is evaluated as a predictive framework whose clinical accuracy is currently limited by the stochastic nature of the biological systems. Additionally, the impact of MOF architectures on nanofiber piezoelectricity is demonstrated, raising questions regarding the sustainability of electroactive effects during framework decomposition. In this way, this review identifies persistent research gaps in material stability and long-term biocompatibility, providing a realistic roadmap for the development of next-generation bioactive wound interfaces.

## Graphical abstract

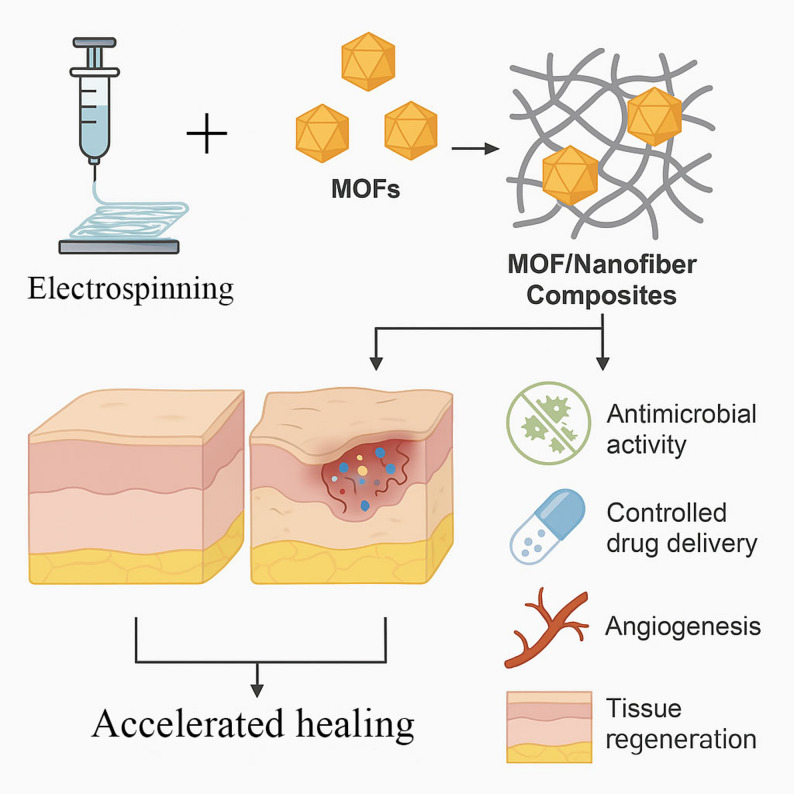

## Introduction

Wound healing is a complex and essential biological process that involves a series of overlapping stages: hemostasis, inflammation, proliferation, and remodeling [1]. Despite advances in medical treatments, chronic and non-healing wounds, such as diabetic ulcers, major burns, and pressure sores continue to pose significant challenges, often leading to prolonged healing times, stubborn infection due to biofilm formation, and substantial healthcare costs [2, 3]. These challenges have driven the need for advanced wound care materials that can actively enhance the healing process.

Nanotechnology has become a pivotal engine in advancing next-generation wound dressings [4, 5]. Among the various nanomaterials, nanofibers have garnered considerable attention due to their remarkable ability to mimic the extracellular matrix of native tissues, thereby fostering an environment conducive to cell adhesion, proliferation, and migration [6, 7]. The integration of nanoparticles within nanofibers has further expanded their utility, facilitating the development of multifunctional wound dressings that not only protect the wound but also actively enhance the healing process [8]. The incorporation of nanoparticles into nanofibers enables a more controlled and sustained release of encapsulated drugs, as the therapeutic agent must traverse two distinct barriers: first, the nanoparticle itself, and second, the nanofibrous matrix [9]. Therefore, the judicious selection of nanoparticles, along with the polymers used in nanofiber fabrication, plays a critical role in determining the release kinetics and overall efficacy of the wound dressing.

Metal Organic Frameworks (MOFs), a class of highly porous and tunable crystalline materials composed of metal ions coordinated to organic ligands, have recently been explored for their applications in wound healing [10–12]. While the therapeutic potential of MOFs is substantial, their clinical relevance is fundamentally governed by the pharmacological and toxicological profiles of their metal centers and organic ligands [13]. Transition metals such as copper and cobalt, although pivotal in stimulating angiogenesis and collagen synthesis, exhibit a narrow therapeutic window; their accumulation is associated with dose- and time-dependent nephrotoxicity and neurotoxicity. Comparative toxicological assessments in zebrafish models have established a distinct hierarchy of acute toxicity, typically ranked as Cu-MOF > Zeolitic Imidazolate Framework (ZIF-8) > Fe-MOF > Zr-MOF. Ultrastructural analysis further revealed that ZIF-8 and Cu-MOF nanoparticles can induce severe mitochondrial fragmentation within the intestinal epithelial and hepatic cells [14].

Standard in vitro assays often underestimate these risks, as many MOFs maintain stability under physiological cell culture conditions. This stability may misleadingly lead to low elution profiles of metal ions or ligands, masking the true cytotoxic potential that would be triggered by the dynamic, acidic microenvironment of a biological wound site. The integration of MOFs into nanofibers addresses these challenges: the polymer matrix provides a protective, flexible housing that prevents MOF leaching and aggregation, while the porous architecture of the MOFs significantly increases the drug-loading surface area and provides sustained drug release [15].

The integration of MOFs into nanofibers can be achieved through various fabrication techniques, with electrospinning being the most widely used [16, 17]. This process allows –to a great extent- a uniform distribution of MOFs within the nanofiber matrix, ensuring that their beneficial properties are effectively utilized. MOF-loaded nanofibers can be designed to provide antimicrobial activity, promote angiogenesis, and deliver drugs or growth factors in response to the specific needs of the wound environment [18]. For example, MOFs containing silver ions have shown a remarkable antimicrobial activity, which is critical in preventing and treating infections in wounds [19, 20]. MOFs based on zinc (Zn-MOFs) have demonstrated a high potential in promoting angiogenesis and reducing inflammation which are key processes in wound healing [21]. Recent advancements in the field have also explored the use of stimuli-responsive MOF-based nanofibers, which can release the loaded therapeutic agents in response to changes in the wound environment, such as pH or temperature variations. This smart release mechanism ensures that the wound is treated with the right dose of therapeutic agents at the right time, optimizing the healing process and reducing the risk of side effects.

Previous reviews explored MOF-incorporated nanofibers in drug delivery [22, 23]. This review examines how the specific loading techniques utilized in MOF-nanofiber fabrication fundamentally dictate the resulting mechanical, physicochemical, and biological performance of the dressing. We pivot the discussion toward the stability-toxicity paradox that is frequently marginalized. By conducting a rigorous assessment of inherent technical constraints, we offer research-driven strategies to mitigate pervasive bottlenecks, such as particle aggregation and non-homogeneous distribution within the polymer matrix.

Furthermore, we demonstrate the realistic limitations of Machine Learning in forecasting in vivo outcomes, specifically addressing how the stochastic variables of the wound microenvironment can undermine the predictive accuracy. Crucially, the influence of MOF architectures on nanofiber piezoelectricity is evaluated; raising pivotal questions regarding the sustainability of electroactive effects as the framework inevitably decomposes under physiological conditions. By bridging these multifaceted research gaps, this work provides a balanced, engineering-centric roadmap for transitioning these scaffolds from idealized experimental models towards future reliable clinical interfaces.

## Electrospinning for biomedical applications: from basics to clinical potential

Electrospinning is a versatile technique widely used for fabricating fine fibers, typically within the nanometer to micrometer range, by employing an electric field to draw a polymer solution or melt into filaments. A standard electrospinning setup consists of three core components: a syringe filled with the polymer solution, a high-voltage power source, and a grounded collector, which may be flat or cylindrical. Upon the application of voltage, the electrostatic forces overcome the surface tension at the droplet tip, leading to the formation of a Taylor cone and the subsequent emission of a polymer jet. As the jet travels toward the collector, it experiences stretching, bending, and solvent evaporation, resulting in the formation of continuous ultrafine fibers [24–26].

Electrospun fibers structurally resemble the extracellular matrix, which plays a crucial role in supporting cell attachment, proliferation, and differentiation. Their porous architecture, high surface-area-to-volume ratio, and ability to incorporate bioactive substances make them highly suitable for various therapeutic uses, including wound dressings, drug delivery platforms, antimicrobial coatings, and tissue engineering scaffolds [27].

Although conventional electrospinning systems are primarily restricted to laboratory-scale production, recent advancements have made the technology more adaptable for clinical and industrial use. For instance, portable electrospinning devices now allow direct application of nanofibers onto patient wounds, enabling personalized treatment at the point of care with minimal contamination risk [28]. Additionally, needleless electrospinning or multi-needle, which generates multiple jets from a free surface (like a rotating cylinder), offers a scalable solution suitable for industrial-level nanofiber production [29, 30].

When compared with more traditional wound management technologies—such as hydrogels, foams, or films—electrospun nanofibers demonstrate notable advantages, including their biomimetic architecture, effective drug encapsulation and release, high oxygen permeability, and adaptability in design and material composition. Nevertheless, challenges such as consistency in production, mechanical durability, and regulatory compliance remain critical barriers [31].

## Overview of MOF categories in biomedical applications

MOFs have emerged as highly versatile materials in the biomedical field due to their tunable porosity and functionalizability. Their structural diversity enables a wide range of applications; from drug delivery and imaging to biosensing and tissue engineering (Table 1).

**Table 1:** Summary of key MOFs used in biomedical research, highlighting their composition, structural characteristics, and specific therapeutic or diagnostic roles

Category	Examples	Key Features	Biomedical Applications	Special Highlight	Ref
Zinc-Based MOFs	ZIF-8, Zn-MOF-74	Biocompatible, pH-responsive, simple synthesis	Drug delivery, antibacterial, cancer therapy	ZIF-8 degrades in acidic environments, enabling targeted drug release.	[32–35]
Iron-Based MOFs	MIL-100(Fe), MIL-101(Fe), MIL-53(Fe)	Biodegradable, magnetic, redox-active	Magnetic resonance imaging, drug delivery, photothermal therapy	Serve as both imaging agents and drug carriers (theranostics).	[36–39]
Zirconium-Based MOFs	UiO-66, UiO-67	Highly stable, large surface area	Cancer drug delivery, gene delivery, photodynamic therapy	Stable in biological fluids, ideal for controlled therapeutic delivery.	[40, 41]
Copper-Based MOFs	HKUST-1, Cu-BTC	Redox-active, catalytic, antibacterial	Antimicrobial wound dressings, biosensing	Releases Cu²⁺ ions for membrane disruption in bacteria.	[42, 43]
Lanthanide-Based MOFs	Eu-MOFs	Luminescent, photostable	Bioimaging, sensing, theranostics	High-resolution imaging and real-time tracking with low toxicity.	[44–46]
Silver-Based MOFs	Ag-carboxylate MOFs	Strong antimicrobial, long-term ion release	Infection control, wound healing	Antibacterial action without external antibiotics.	[47, 48]
Cobalt-Based MOFs	Co-BTC, Co-MOF-74, Co-MOF-199	High stability, magnetic properties, and tunable porosity	Antibacterial agents, drug delivery systems, biosensors, and imaging agents	Beneficial effect depends on slow release of Co²⁺ ions	[49, 50]

ZIF-8: Zeolitic Imidazolate Framework, MIL: Materials of Institute Lavoisier, UiO: University of Oslo, BTC: benzene-1,3,5-tricarboxylic acid, HKUST-1: Hong Kong University of Science and Technology

## Common MOFs/nanofibers composites

The integration of MOFs with electrospun nanofibers offers a versatile and innovative approach to enhancing the functionality of nanofibrous scaffolds for biomedical applications. Owing to nano-in-nano effect, these MOF/nanofiber composites combine the high surface area, tunable porosity, and controlled drug release capabilities of MOFs with the flexibility and structural features of nanofibers, enabling multifunctionality such as a dual drug delivery and targeted wound therapy [9]. Beyond drug release, MOF incorporation can significantly improve the mechanical performance of nanofibers. Acting as nanofillers, MOFs can reinforce the fibrous matrix by increasing tensile strength, elasticity, and structural stability which are essential features for wound dressings and load-bearing biomedical materials [51].

MOFs can be incorporated into the electrospun nanofibers through two main methods: blending and in situ growth [52]. The selection of a specific strategy should be guided by the desired functional outcome. For example, the blending or the doping method—where MOF particles are physically mixed with the polymer solution before electrospinning—is often preferred for applications requiring sustained drug release, especially when rapid ion release (e.g., from copper-based MOFs) may lead to toxicity concerns [53]. In contrast, in situ growth, which involves synthesizing MOFs directly on the surface of pre-formed nanofibers, is beneficial when intimate MOF-biomaterial interactions are required. This method can enhance surface properties such as wettability and reactivity. For instance, in situ growth of ZIF-8 on polycaprolactone (PCL)/lignin nanofibers could significantly improve the hydrophilicity of the composite which is an important trait for wound healing scaffolds [54].

The subsequent sections will explore the fabrication techniques and biomedical performance of MOF/nanofiber composites, with a focus on how specific synthesis strategies influence the mechanical, physicochemical, and therapeutic properties.

### Zeolitic Imidazolate Framework (ZIF-8)- loaded nanofibers

**Fig. 1:**
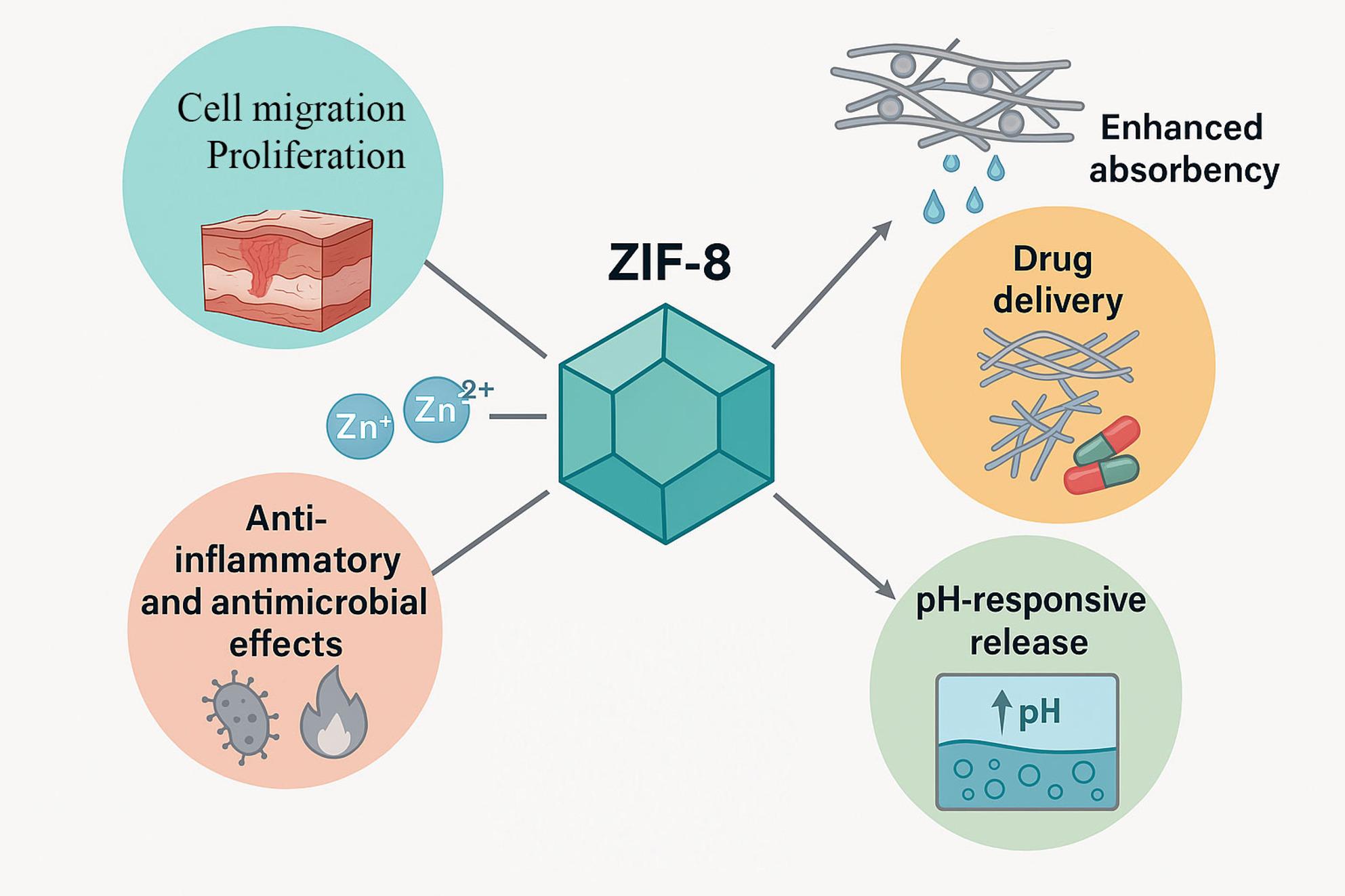
Schematic illustration of the characteristics of ZIF-8-loaded nanofibers

ZIF-8 synthesis is facile since the coordination between Zn ions and 2-methyl imidazole, is rapid and does not require the solvothermal treatment. Furthermore, ZIF-8 is commonly employed in creating biomaterial composites for wound healing because of its large surface area, high capacity for loading different drugs, and the release of zinc ions which have powerful anti-inflammatory and antimicrobial effects (Fig. 1) [55–57]. Additionally, the pH-responsive release of the loaded drugs from ZIF-8 is highly significant [58, 59].

ZIF-8 embedded in nanofibers can potentially provide sequential drug release pattern which suits the wound healing stages. Yin Lei et al. loaded dimethyloxalylglycine in ZIF-8 and electrospun it with gelatin-PCL. As the nanofibers degrade, the ZIF-8 nanoparticles decompose, sequentially releasing bactericidal zinc ions and angiogenic dimethyloxalylglycine molecules [60]. ZIF-8@nanofibers enables the simultaneous incorporation of multiple drugs within the nanofiber matrix and the ZIF-8 matrix, allowing them to serve different purposes. For example, in the case of naringenin @ZIF-8@nanofibers, it demonstrated antibacterial properties by releasing Zn ions and naringenin in a pH responsive pattern, while the carbon quantum dots present in the NF contributed to a photodynamic antibacterial effect [61].

The inclusion of ZIF-8 in the nanofibers could potentially improve the mechanical properties and biostability of the dressing. Studies showed that incorporating ZIF-8 into the 3D nanofibrous network of polylactic acid nanofibers/hydrogel composite increased the stretchiness and tensile strength of the composite [51]. In terms of improving the stability of the nanofibers, it was shown that the natural positive charge of Zn in ZIF-8 can electrostatically bind with other bioactive materials, such as the bioactive glass in the nanofibrous matrix. This significantly enhanced the stability of the bioactive glass in the matrix. The gradual release of Zn, Ca, and Si ions considerably enhanced the expression of growth factors and promoted the regeneration of skin [62]. Additionally, the in-situ growth of ZIF-8 on the surface of nanofibers could significantly enhance the rapid absorption of wound exudates [63].

**Fig. 2:**
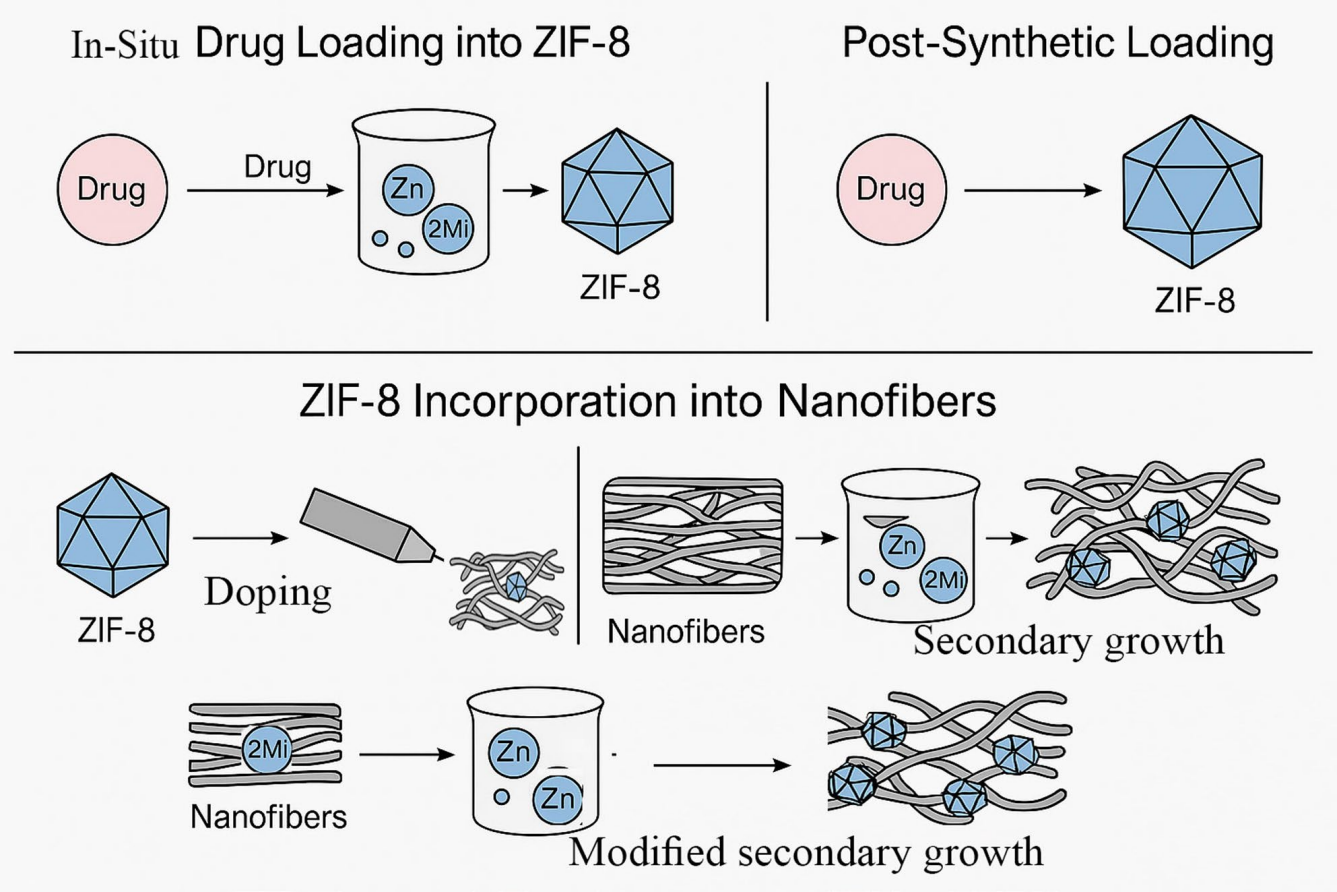
Schematic figure showing the possible methods for drug loading and the incorporation of ZIF-8 into nanofibers, 2Mi: 2-methyl imidazole

Drugs can be loaded into ZIF-8 using two common methods. The first method involves in situ loading during the coordination process between Zn and 2-methyl imidazole (2Mi). On the other hand, the second method requires adding the drug solution to ZIF-8 after its formation. To form ZIF-8/nanofiber composite, ZIF-8 can be incorporated into the nanofibers using three methods (Fig. 2):

- ZIF-8 synthesis followed by mixing with the spinning solution and electrospinning (doping or blending).
- Fabrication of the nanofibers followed by impregnation in Zn and 2Mi (Secondary growth).
- Electrospinning of nanofibers containing the ligand (2Mi) followed by impregnation in Zn solution (Modified secondary growth).

The selection between blending and in situ (secondary) growth is primarily dictated by the physicochemical properties of the MOF and the sensitivity of the therapeutic payload. The blending or the doping method, characterized by blending pre-synthesized MOFs into the spinning solution, is the most straightforward approach, but is highly dependent on MOF size compatibility. Large MOF particles (> 500 nm) can cause needle clogging or create structural defects that compromise the mechanical integrity of the fibers. Furthermore, this method is unsuitable for pH-sensitive MOFs like ZIF-8 if the polymer solution is acidic (pH < 5), as premature degradation can occur during the spinning process [64]. However, blending is often preferred for hydrophilic drugs where a sustained release is desired, as the polymer matrix acts as an additional diffusion barrier.

In contrast, in situ growth—including secondary and modified secondary growth—allows for the decoration of the nanofiber surface with high densities of MOF crystals. This method is particularly advantageous for heat-sensitive or fragile drugs that might not survive the high voltages or organic solvents used in the electrospinning, as they can be loaded into the MOFs under milder aqueous conditions after the fiber scaffold is already formed. While in situ growth enhances surface properties like wettability and rapid exudate absorption, it often leads to a higher drug loading capacity and a faster release profile compared to doped systems, as the MOFs are directly exposed to the wound environment. For instance, the study by Jun Yang et al. revealed different results in drug loading capacity between ZIF-8 crystals embedded in the nanofibers and crystals formed on the surface of the nanofibers after the electrospinning process. The research demonstrated that nanofibers doped with ZIF-8 showed a lower gentamicin loading efficacy compared to the loading of ZIF-8 secondary grown on the nanofiber surface. Consequently, it was shown that the antibacterial effect of gentamicin@ZIF-8 loaded nanofibers prepared by the secondary growth method was superior to the doped preparation [65]. Therefore, if the goal is rapid antimicrobial action or surface-active signaling, in situ growth is the superior choice, whereas doping is better suited for long-term structural reinforcement and steady drug delivery.

### Cu-MOF loaded nanofibers

**Fig. 3:**
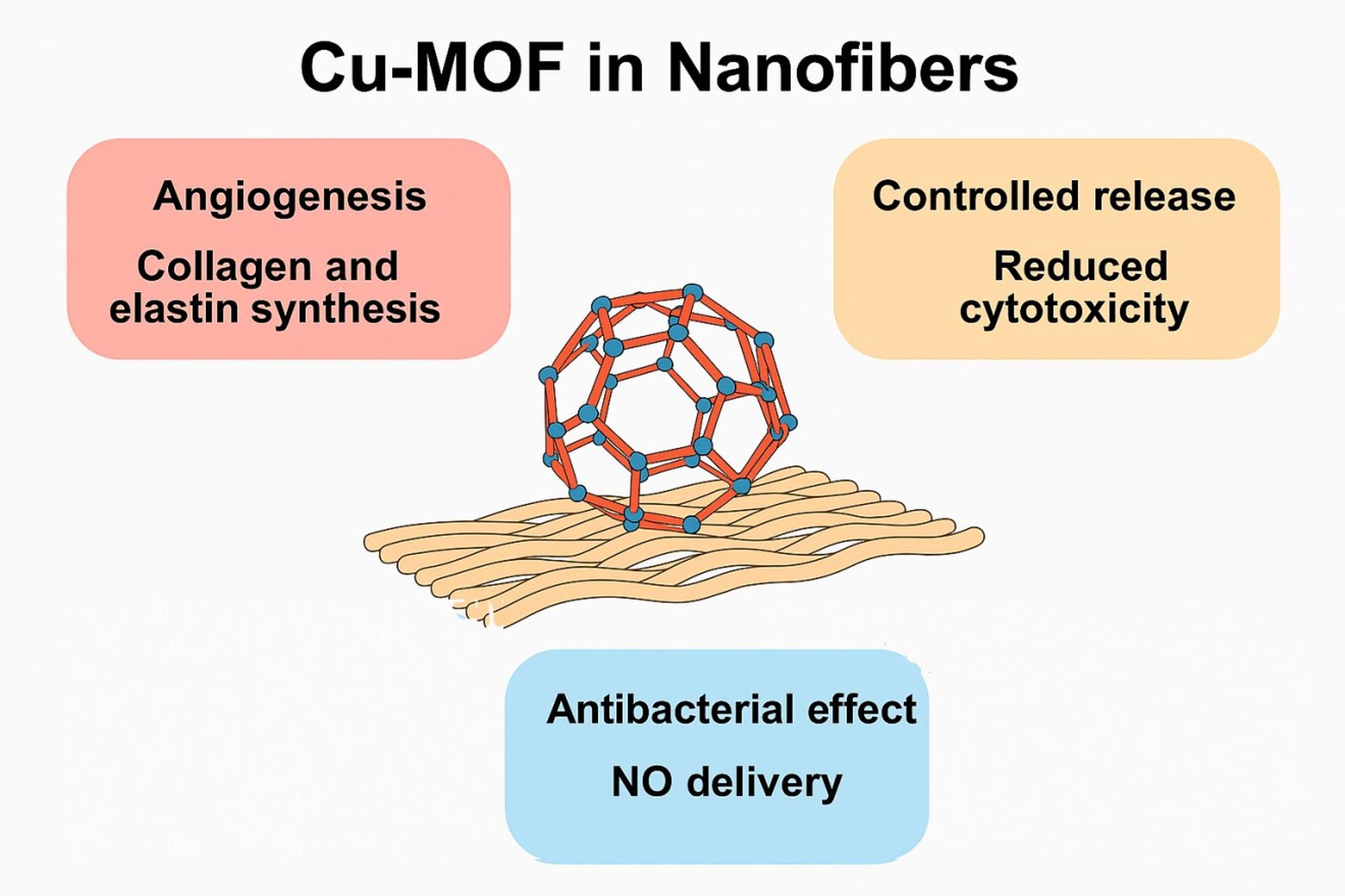
Schematic presentation showing the possible functional properties of nanofibers loaded by Cu-MOF, NO: nitric oxide

Cu is recognized as a vital element for numerous metabolic processes for both the animals and plants. Its applications in formulations like hydrogels, thin films, and copper oxide are extensive, particularly in the realms of bone and skin tissue engineering [66–68]. This widespread use is due to its ability to enhance the angiogenic properties, promote collagen and elastin synthesis while also providing robust antibacterial effects [69]. Nonetheless, the necessity for controlled release is paramount to mitigate Cu’s potential toxicity [70]. Cu-based MOFs are commonly utilized for this purpose [71]. Cu coordinated in MOFs often leads to a more controlled release pattern, especially when incorporated into a hydrogel formulation [72, 73]. Compared to ZIF-8, Cu-MOFs provide enhanced angiogenesis, but require a more precise control to avoid cytotoxicity. However, it is imperative to discuss the Cu-MOF loaded nanofibers to elucidate the influence of the nanofibrous matrix on the release of Cu and other encapsulated drugs from Cu-MOF, as well as to assess how the integration of Cu-MOF affects the functional properties of the nanofibers (Fig. 3).

The inherently large pore size of MOFs, particularly Cu-MOFs, facilitates the multiple co-loading of functional biomaterials into nanofibers. For instance, a multifunctional smart dressing was developed by incorporating glucose oxidase and carbon quantum dots into Cu-MOFs, followed by embedding them in a nanofibrous matrix. This dressing was capable of monitoring wound pH through the fluorescent indication provided by the quantum dots. Additionally, glucose oxidase converted endogenous glucose into hydroxyl radicals, exerting a strong antibacterial effect, which was further potentiated by the slow release of copper [74].

Incorporation methods into nanofibers heavily rely on the morphological characteristics of the fabricated Cu-MOFs. Certain Cu-MOFs have large dimensions that necessitate specialized techniques to create Cu-MOF@nanofiber composites for wound healing applications. For example, micro-structured, donut-shaped copper coordinated with nicotinate has proven to be particularly suitable for blending with 2,2,6,6-tetramethylpiperidine-1-oxyl -oxidized bacterial cellulose nanofibers to form a three-dimensional nanofibrous composite. The synergy between nicotinic acid, released copper, and the 3D structure of the nanofibrous scaffold has been shown to significantly enhance wound healing efficacy [75].

Hong Kong University of Science and Technology (HKUST-1) is recognized as the most readily embeddable Cu -based MOF in electrospun nanofibers due to its adjustable small dimensions. Incorporating HKUST-1 into chitosan-based nanofibers significantly enhances their inherent antibacterial efficacy, achieving a 99% eradication rate against both *E. coli* and *S. aureus*. Furthermore, the controlled release of copper from HKUST-1@nanofiber does not compromise the cytocompatibility of the chitosan-based nanofibers [76]. Introducing a co-ligand like folic acid alongside trimesic acid (the main ligand) during the fabrication of HKUST-1 enhances the compactness and stability of Cu-MOF@NF in aqueous environments, leading to prolonged release of copper and folic acid. Interestingly, incorporating silver nanoparticles into HKUST-1 notably reduced the MOF particle size from 470 nm to 95 nm, which in turn enhanced the antimicrobial activity and improved loading efficiency into electrospun chitosan/polyvinyl alcohol/hyaluronic acid nanofibers [77]. The electrospun pectin nanofibers loaded with Folic-HKUST-1 demonstrate an extended cumulative release of copper lasting over nine days. This prolonged release contributed to a sustained antibacterial efficacy, reduced cytotoxicity, and improved mechanical properties [78]. It was noticed that the loading of HKUST-1 and fluoride based HKUST (F-HKUST) into a highly biocompatible gelatin-based nanofibers significantly reduced the toxic effect of the released Cu or fluoride from the nanofibers [79].

Nitric oxide-(NO) releasing nanofibers have been extensively studied for their ability to enhance the wound healing process. The released NO plays a crucial role in modulating the expression of growth factors essential for neo-angiogenesis, as well as promoting cell migration, adhesion, and proliferation, in addition to providing an antibacterial effect. Direct loading of NO into nanofibers is generally infeasible due to its short lifetime. Instead, nanofibers are loaded with molecules such as sodium nitroprusside and S-nitrosoglutathione, which can indirectly decompose to produce NO [80, 81]. Notably, it was discovered that HKUST-1 can be directly loaded with gaseous NO, particularly following the incorporation of a secondary amino group. Integrating HKUST-1 into hydrophobic electrospun polycaprolactone nanofibers resulted in a sustained NO release extending over 14 days [82].

### Ag-MOF loaded nanofibers

Silver nanoparticles sequestered within electrospun nanofibers have been extensively documented for their capacity to provide a sustained Ag⁺ ion release, thereby extending the duration of the antimicrobial activity in clinical wound management [83–85]. The versatility of the nanofiber scaffold further allows for the co-incorporation of auxiliary therapeutic agents, such as lavender oil, to achieve synergistic effects between pathogen eradication and fibroblast proliferation [86]. However, while silver nanopartilces represent a mature technology, Ag-MOFs offer a superior architectural advantage; their intrinsic porosity and expansive surface area significantly facilitate higher loading capacities for secondary bioactive molecules. A notable example is the in-situ growth of Ag-MOFs onto 3D polycaprolactone/gelatin nanofibers for the co-delivery of curcumin [87].

Despite these advancements, the stability-toxicity paradox remains a critical hurdle for Ag-MOF clinical translation. As Ag typically exists as a monovalent cation with a high reactivity, the framework often lacks the structural robustness required for a controlled elution. To mitigate the risk of premature leaching and a localized toxicity, researchers have explored the encapsulation of Ag-MOFs within more chemically resilient frameworks, such as ZIF-8. When integrated into chitosan/cellulose nanofibers, such composites demonstrated a 52–300% increase in the antibacterial potency through an improved stability and a synergic effect [88]. Consequently, the conceptual role of the nanofiber matrix must evolve from a passive vehicle to an active regulatory sheath that governs the diffusion kinetics of Ag ions [89–91]. Future investigations must prioritize the metabolic fate of these degraded Ag -organic ligands to ensure that the immediate antimicrobial success does not inadvertently impede the desired long-term tissue regeneration.

### Zr-MOF loaded nanofibers

**Fig. 4:**
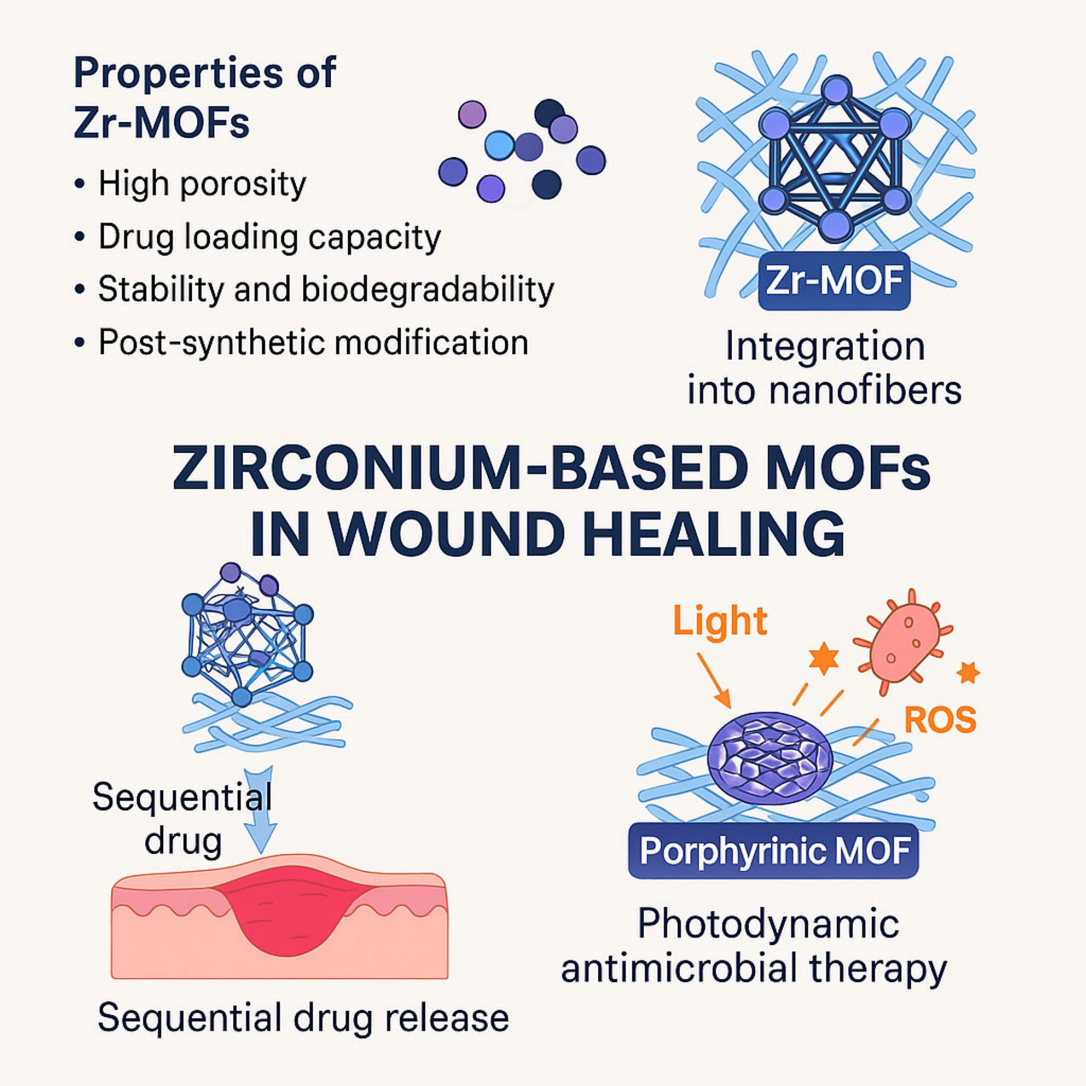
Schematic illustration showing the characteristics of Zirconium-based MOFs and Zirconium MOFs loaded nanofibers

Zr-MOFs, particularly the University of Oslo (UiO) and Porous Coordination Network (PCN) series, are recognized as robust candidates for drug delivery due to their exceptional porosity, high drug-loading capacity, and superior hydrolytic stability (Fig. 4) [92–95]. However, a critical evaluation of the reported literature reveals a fundamental functional trade-off: unlike their Zn or Cu counterparts, Zr-MOFs are predominantly biologically passive and lack intrinsic antimicrobial or pro-angiogenic signaling properties. Consequently, their clinical utility is derived almost exclusively from their performance as high-surface-area reservoirs for secondary therapeutic payloads.

For instance, while the integration of UiO-66-NH₂ into polyvinyl alcohol nanofibers improved water stability and enabled the extended release of levofloxacin, the therapeutic outcome was entirely dependent on the encapsulated drug rather than the framework itself [96]. While this structural robustness effectively prevents the burst release toxicity, it introduces significant questions regarding the metabolic fate of the scaffold. The slow degradation kinetics of Zr-IV coordination bonds may lead to the prolonged presence of crystalline residuals within the regenerating tissue which is a critical factor seldom addressed in current literature. Future research must evaluate whether this persistence interferes with long-term extracellular matrix remodeling.

Furthermore, the stability of Zr-MOFs has made them an ideal platform for porphyrinic MOFs (Fig. 4) used in photodynamic antimicrobial therapy [97]. Frameworks such as PCN-224 utilize the Zr-center to stabilize the photosensitizers, enabling the generation of reactive oxygen species upon irradiation [98, 99]. While these systems, such as PCN-224/polycaprolactone composites, show a remarkable efficacy against multi-drug-resistant pathogens, critical consideration must be given to the light-penetration depth required for activation. The efficacy of such photoactive membranes is inherently limited by the opacity of the dressing and the depth of the wound, suggesting that while Zr-based porphyrinic MOFs are chemically superior, their clinical application may be restricted to the superficial infections unless coupled with more penetrative light sources or bimetallic enhancements [100, 101].

## Potential MOFs/nanofibers candidates

While Zn and Cu-based MOFs such as ZIF-8 and HKUST-1 remain the most extensively investigated systems for MOF–nanofiber wound dressings, a growing attention has recently shifted toward iron- and cobalt-based MOFs due to their unique biological activities. However, their clinical translation requires a careful benchmarking against mature MOFs in terms of the anticipated antibacterial efficacy, cytocompatibility, and wound healing performance.

### Iron-based MOFs versus ZIF-8 and HKUST-1 nanofiber composites

Iron-based metal–organic frameworks (Fe-MOFs), including Materials of Institute Lavoisier (MIL-100(Fe), MIL-101(Fe), and MIL-53(Fe), have emerged as potent antibacterial agents primarily due to their Fenton-like catalytic activity, which enables the generation of reactive oxygen species. This mechanism confers Fe-MOFs with an exceptionally high antibacterial efficacy, frequently exceeding 99% eradication of drug-resistant bacteria, including *Staphylococcus aureus* and *MRSA*, outperforming ZIF-8 and comparable to HKUST-1 in infected wound models [102–104]. The previously mentioned studies regarding ZIF-8-loaded nanofibers typically maintain high cytocompatibility (> 90% cell viability) while achieving a moderate antibacterial activity through Zn²⁺ release and membrane disruption. Fe-MOFs demonstrate a superior bactericidal performance, but their rapid degradation under physiological or mildly acidic wound conditions can lead to excessive Fe²⁺/Fe³⁺ release, potentially inducing an oxidative stress and prolonged inflammation [105].

Embedding Fe-based MOFs within nanofibrous matrices has emerged as a critical strategy to alleviate their inherent limitations. Electrospun polymer networks function as effective diffusion barriers, moderating MOF degradation and enabling a sustained reactive oxygen species-mediated antibacterial activity without inducing an excessive damage to the surrounding tissues. For example, a previous study demonstrated that Fe-MOF incorporation into polyacrylonitrile nanofibers supported acceptable cell proliferation up to a polymer:10% Fe-MOF composition, whereas higher Fe-MOF loadings resulted in a marked decline in the cellular viability [106]. In vivo investigations further revealed that Fe-MOF–integrated nanofibers significantly accelerated wound healing, particularly in infected wound models. Nevertheless, when compared with ZIF-8-based dressings, Fe-MOF composites require a more stringent control over dosage and release kinetics to minimize cytotoxic effects, highlighting the indispensable role of nanofiber encapsulation in expanding their therapeutic window [107].

### Cobalt-based MOFs versus ZIF-8 and HKUST-1 nanofiber composites

Cobalt-based MOFs (Co-MOFs), such as ZIF-67 and Co-MOF-74, offer a fundamentally different therapeutic advantage by actively promoting angiogenesis and tissue regeneration through cobalt-mediated stabilization of hypoxia-inducible factor-1α [108]. Compared to ZIF-8, Co-MOFs directly influence the proliferative and remodeling phases of wound healing in a greater way. However, cobalt ions possess a narrow therapeutic window, and uncontrolled Co²⁺ release may result in cytotoxicity and inflammatory complications [109]. Compared to HKUST-1, cobalt-based MOFs do not only demonstrate a robust antimicrobial activity, but also uniquely contribute to the angiogenesis, offering a dual therapeutic advantage in the wound healing applications [110]. Nanofiber integration has been shown to be an effective strategy for modulating cobalt ion release. ZIF-67 embedded within polyvinyl alcohol /chitosan nanofibers maintained a high fibroblast viability (> 85%) while achieving up to 90% antibacterial efficiency against *E. coli* and *S. aureus* [18].

To provide a structured synthesis of the diverse findings across various MOF/nanofiber architectures, Table 2 offers a comprehensive comparative evaluation of representative systems discussed in the preceding sections. This consolidated overview encompasses critical parameters, ranging from the selection of the polymer matrix and MOF incorporation strategies to the specific therapeutic payloads and their associated biological outcomes.

**Table 2:** Summary of various MOF/nanofiber composites for wound healing, highlighting polymer matrices, fabrication strategies, and therapeutic outcomes

MOF	Polymer matrix	Method of MOF incorporation	Drug loaded	Drug loading technique	Antibacterial efficacy	In vivo results	Ref.
ZIF-8	hierarchical micro/nanofibrous poly L-lactic acid	Blending	Curcumin	In-situ during ZIF-8 formation		Reduced the inflammatory response of diabetic wounds, reduced the oxidative stress. Promoted epidermal regeneration, angiogenesis and collagen deposition	[111]
ZIF-8	Polycaprolactone	Blending	Rose Bengal	In-situ during ZIF-8 formation	Photodynamic mediated complete destruction of *Staphylococcus aureus* and *Escherichia coli*	Reduced bacterial bioburden on the wound and accelerated the healing process	[112]
ZIF-8	Polycaprolactone /Sulfonated chitosan	Blending	-	-	*Effective against Staphylococcus aureus* and *Escherichia coli* with inhibition of 32.3 mm and 31.2 mm, respectively		[113]
ZIF-8	Polyvinyl alcohol /Chitosan/Hyaluronic acid	Blending	-	-	Proficient antimicrobial activities against *Bacillus cereus*,* Listeria monocytogenes*,* Staphylococcus aureus*,* Salmonella organisms*,* Escherichia coli*,* Pseudomonas aeruginosa*,* Candida tropicals*		[114]
ZIF-8	Polycaprolactone	Blending	Curcumin	After the formation of ZIF-8	Inhibition zone of 14 ± 0.65 and 8 ± 0.72 mm for *E. coli and S. aureus*, respectively		[115]
ZIF-8	Poly lactic acid	Modified secondary growth	Indocyanine green/Curcumin	In-situ during ZIF-8 formation on the surface of nanofiber	Stimuli-responsive release with bactericidal rates of more than 99% Photothermal/ Photodynamic antimicrobial effect	Significant reduction of bacterial bioburden at the wound site and more than 92% reduction of the wound size after 14 days	[116]
ZIF-8	Polyacrylonitrile	Secondary growth	Portulaca oleracea extract	After the formation of ZIF-8 on the surface of nanofiber	Inhibition zone of 18.91 ± 0.69 mm against *S. aureus and 15.70 ± 2.29 mm against E. coli*		[117]
ZIF-8	Polyvinyl alcohol	Blending	Vancomycin	In-situ during ZIF-8 formation	The antibacterial effect of was 99% for *E. coli* and *S. aureus*		[118]
HKUST-1	Chitosan/ polyvinyl alcohol	Blending			The kill ratios against both bacteria were over 99.0% for both *E. coli* and *S. aureus*	Wound closure was 99.1% by the 12th day	[76]
HKUST-1	Pectin	Blending	Folic acid	In-situ during HKUST formation	The kill ratios against both bacteria were 100% for both *E. coli* and *S. aureus*		[78]
HKUST-1	Polycaprolactone	Blending	NO	After the formation and modification of HKUST by 4-(Methylamino) pyridine		Enhanced angiogenic effect by the dual release of Cu ions and NO	[82]
HKUST-1	Polyvinyl alcohol /gelatin	Blending	Fluoride	In-situ during HKUST formation	The antimicrobial efficacy tests showed the highest growth reduction percentages in *S.aureus* (99.72 ± 0.78%), *Klebsiella pneumoniae* (97.76 ± 0.44%),and *Candida parapsilosis* (94.50 ± 0.75%)		[79]
Ag-MOF	Polylactic acid	Blending			High potency against *E. coli*, *Pseudomonas aeruginosa S. aureus* and *Mycobacterium smegmatis*	Nearly complete wound healing within 14 days. Antibacterial mechanism involved Ag+ leaching and reactive oxygen species production	[89]
Ag-MOF	Polycaprolactone /gelatin	Secondary growth	Curcumin	After the formation of MOF	Nearly complete microbial eradication (99%) against *E. coli* and *S. aureus*	Complete wound healing on day 12	[87]
Zr-basedUiO-66-NH2	Polyvinyl alcohol	Secondary growth	Levofloxacin	During the nucleation of MOF on the surface of the nanofibers	Bactericidal with the efficiency > 99.9% against *E. coli* and *S. aureus*	Nearly complete healing within 14 days	[96]
Fe-MOF	Polycaprolactone	Blending				did not provoke any inflammatory responses	[119]
Fe-MOF	Polycaprolactone /gelatin/glucose	Anchoring on the surface of the nanofibers		Coupling of glucose oxidase with Fe-MOF by amide bond	Shrinking of the surface of MRSA by the contact with the nanofibrous matrix	Eradication or MRSA from the infected wound by the catalytic hydroxyl production and achieved healing comparable to the commercial silver dressing	[120]
Co-MOF (ZIF-67)	Chitosan/polyvinyl alcohol				Antibacterial activity up to 90%	Marked reduction in inflammation and enhanced skin remodeling followed by complete healing	[18]

## Limitations of MOF/nanofiber composites in wound healing

### Technical and fabrication challenges

The transition from laboratory-scale MOF synthesis to the reliable production of MOF/nanofiber membranes is fraught with rheological and electrostatic hurdles. Achieving a uniform dispersion of crystalline MOFs within a polymeric jet requires more than simple mixing; it necessitates a fundamental understanding of the interfacial energy between the MOF surface and the spinning solvent. As summarized in Table 3, the specific technical bottlenecks—ranging from needle clogging due to particle size mismatch—require targeted research-based interventions. Identifying these constraints is essential for shifting the research focus toward more stable and reproducible fabrication protocols.

**Table 3:** Summary of technical bottlenecks in MOF/nanofiber electrospinning: root causes and research-validated strategies for process optimization

Technical Challenge	Root Cause	Research-Based Solutions & Strategic Criteria	Ref.
Needle Clogging and Jet Interruption	MOF particle size exceeds the threshold	• Prioritizing downsized nanocrystals (< 200 nm) • Transitioning to syringeless or coaxial electrospinning • Implementing in situ growth or secondary crystallization post-spinning	[121, 122]
Non-homogeneous Particle Dispersion	High surface energy of MOF crystals leading to sedimentation or agglomeration in the spinning dope	• Employing intermittent sonication or high-shear homogenization during the process • Developing portable/in situ spinnerets that allow for continuous agitation of the dispersion	[74, 123]
Poor Interfacial Adhesion	Physicochemical mismatch between hydrophilic MOF surfaces and hydrophobic polymer matrices	• Performing surface modification with specific functionality that can coordinate MOF • Integrating cross-linking agents to chemically anchor the MOFs to the polymer backbone	[124]
Uncontrolled ‘Burst’ Release	Weak physical entrapment or surface-localized clusters of MOFs	• Utilizing core-shell (coaxial) architectures to sequester MOFs in the fiber core	[125]

### Biological limitations

Although MOF/nanofiber composites hold a considerable promise for enhancing wound healing outcomes, several critical limitations must be carefully addressed prior to their translation into clinical practice. A significant concern lies in the potential cytotoxicity associated with the release of metal ions from MOFs, particularly those based on transition metals such as Cu, Co, and Zn. While these ions are known to exert beneficial biological effects in trace amounts, their uncontrolled or rapid release can provoke oxidative stress and damage to healthy tissues, thereby narrowing their therapeutic window within wound microenvironments [126, 127].

It was demonstrated that the cytotoxicity profile of HKUST-1 toward fibroblasts and keratinocytes is comparable to that of copper sulfate, a phenomenon largely attributed to the rapid decomposition of HKUST-1 crystals in protein-rich media, which results in an uncontrolled Cu²⁺ release. Remarkably, when HKUST-1 was incorporated into a poly (polyethylene glycol citrate-co-N-isopropylacrylamide) matrix, its cytotoxic effects were substantially attenuated. Even at high concentrations (1 × 10⁻³ M), the composite exhibited a minimal toxicity, no detectable induction of apoptosis, and promoted the highest level of cell migration, underscoring the critical role of polymer encapsulation in modulating Cu bioavailability and enhancing cellular compatibility [128]. Consequently, optimizing the polymeric nanofiber matrix to precisely regulate ion release—maximizing therapeutic benefit while preventing toxicity—remains a critical and challenging aspect in the design of MOF-based wound dressings.

A significant limitation of many MOFs—most notably ZIF-8—is their susceptibility to hydrolytic degradation and framework instability under the acidic conditions commonly associated with chronic wounds [129]. Such instability can lead to a premature drug release and deterioration of the mechanical integrity of the nanofibrous matrix. For example, it has been demonstrated that pre-coating ZIF-8 nanoparticles with hydroxyapatite prior to their incorporation into poly(L-lactic acid) scaffolds effectively preserves nanoparticle integrity. In this system, the cumulative release of Zn ions from the scaffold was reduced by 65.3% after 28 days of immersion compared with the scaffold without surface modification [130]. Despite its effectiveness, this additional coating step introduces a considerable processing complexity. Moreover, common strategies used to integrate MOFs into nanofibers—such as in situ crystallization or blending—often impose substantial challenges in terms of scalability and manufacturing cost, thereby limiting their practical implementation in large-scale biomedical production [131].

Regulatory uncertainties further compound these issues. Given the relatively recent emergence of MOF-based biomaterials, comprehensive long-term safety profiles, including data on biodegradation byproducts, immunogenicity, and chronic exposure, are still lacking. Standardized evaluation frameworks are essential to ensure an acceptable consistency in their performance and to satisfy safety requirements for the needed clinical approval [132].

A critical evaluation of the current literature reveals that while MOF/nanofiber composites are frequently presented as idealized solutions, their clinical feasibility is often contingent upon variables that remain insufficiently characterized. For example, the chemical stability of the MOF-polymer interface in the presence of proteolytic enzymes and acidic wound exudates is a persistent research gap. Furthermore, researchers must carefully consider the trade-off between surface-exposed MOFs for immediate antimicrobial ‘bursts’ and embedded MOFs for a sustained, long-term therapeutic delivery.

## Future perspectives

### Machine leaning (ML) and the future of MOFs/nanofibers composites

**Fig. 5:**
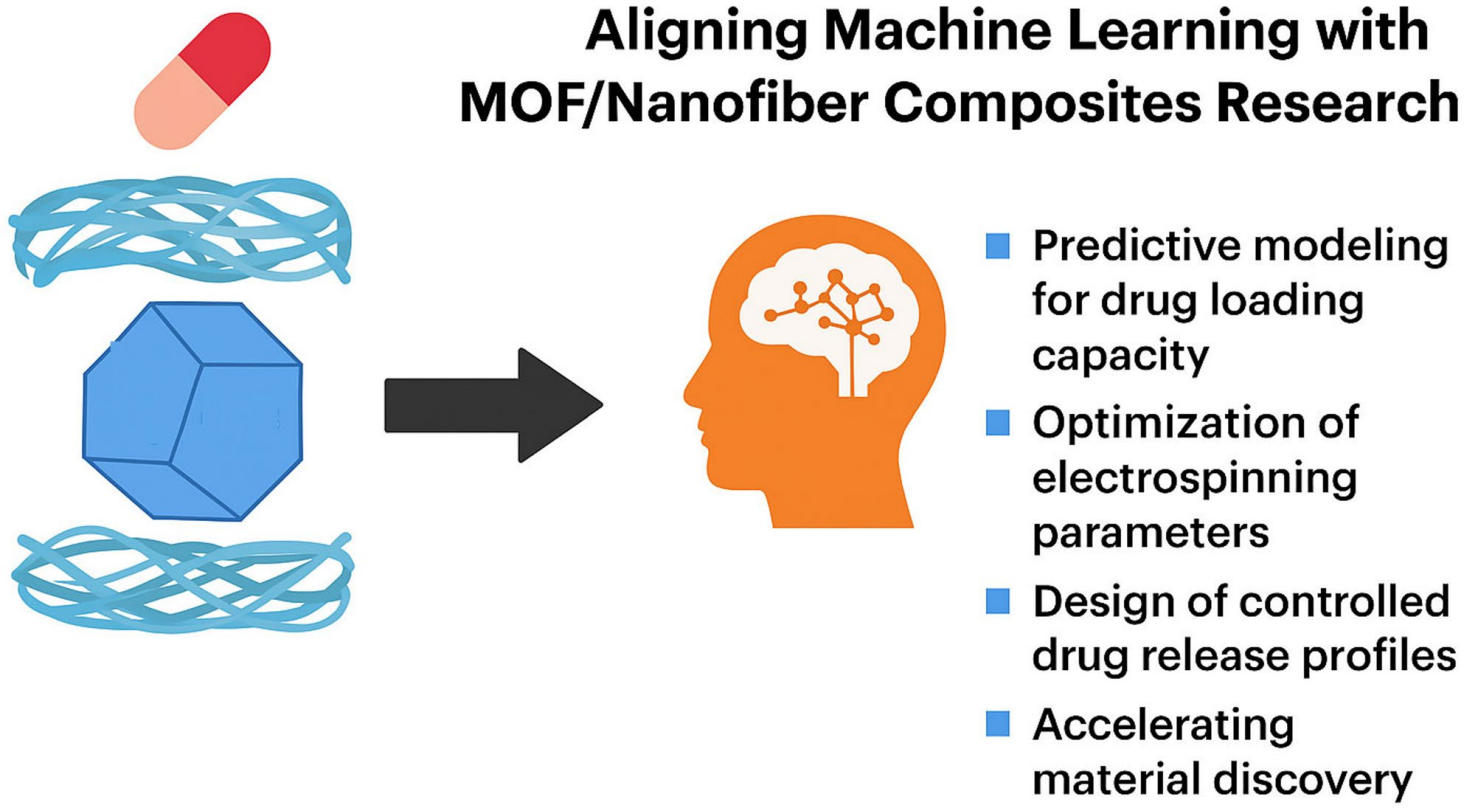
Possible machine learning contrinution to MOFs/nanofibers composites

The utilization of ML into the development of MOF/nanofiber composites holds a great potential for advancing drug delivery systems (Fig. 5). ML techniques can uncover intricate patterns linking the structural features of MOFs, nanofiber morphology, and drug characteristics, allowing for an accurate prediction of drug loading capacities. In one study, algorithms such as support vector regression and random forest were employed to estimate ibuprofen loading across various MOFs, taking into account factors like organic linkers, metal centers, functional groups, surface area, and pore volume. These models achieved a notable coefficient of determination (R²) of 0.76. A similar framework could be leveraged to predict the behavior of different drugs within MOF-based nanofiber systems, enabling a smarter material selection and process optimization [133].

Electrospinning, a key method for fabricating nanofibers, involves several tunable parameters that influence the outcomes like fiber diameter and porosity [134]. ML has proven useful in navigating this complex design space, offering a systematic alternative to the traditional trial-and-error approaches [135]. Recently, it was demonstrated how ML applications have streamlined electrospinning workflows, loading efficacy, and nanofiber diameter [136]. For example, ML studies on curcumin loaded nanofibers demonstrated that the optimal outcomes such as the smallest fiber diameter, and highest encapsulation efficiency were achieved when the polymer had a molecular weight between 100 and 150 kDa, a curcumin concentration of 10–15 wt%, and a polymer density ranging from 1.2 to 1.5 g/mL [137]. These strategies could be particularly valuable when applied to MOF-loaded nanofibers, where fine-tuning is critical for achieving targeted functionality [138]. Moreover, ML can be employed to tailor the drug release profiles by learning from the data related to the polymer types [139]. These insights enable a predictive modeling of release kinetics, paving the way for the design of nanofibrous scaffolds that deliver drugs in a controlled, sustained manner, thereby boosting the therapeutic outcomes.

Historically, the deployment of ML algorithms in MOF synthesis has been largely confined to catalytic applications, specifically the structural optimization of frameworks to maximize the production of reactive oxygen species [140]. For instance, predictive modeling has been extensively utilized to engineer Fe-MOFs that enhance reactive oxygen species yield via the Fenton reaction. However, translating these materials into tissue engineering scaffolds necessitates a more nuanced computational approach [141]. In a clinical context, the objective shifts from maximizing output to achieving a homeostatic balance; algorithms must be designed to optimize both the fabrication parameters and the polymer matrix selection to ensure that reactive oxygen species generation provides potent antimicrobial efficacy without exceeding the threshold for oxidative tissue deterioration.

Despite this transformative potential, several intrinsic limitations hinder the deployment of ML in clinical wound healing. A primary technical hurdle is the ‘small data’ problem; most published studies on MOF/nanofiber composites report a limited number of experimental iterations, which can lead to a model overfitting—a state where the algorithm performs exceptionally on training data, but fails to generalize to the variability of real-world samples. The challenge is further compounded when transitioning from laboratory settings to living systems, where numerous stochastic factors can alter ML-based predictions. In vivo drug delivery kinetics are significantly influenced by many physiological variables such as the wound exudate, pH fluctuations, and the proteolytic enzymes that accelerate scaffold degradation. These ‘biological noise’ factors are rarely captured in the static datasets used for training the model.

### Electroactivity of MOFs/nanofibers composites and wound healing

Electroactive nanofibers have recently garnered a significant interest for tissue engineering applications, particularly in wound healing [142]. This interest stems from the enhanced healing observed in electrically stimulated wounds, attributed to improved cell migration, adhesion, and proliferation on nanofibrous scaffolds [143]. Certain nanofibers, such as Polyvinylidene fluoride and poly L-lactic acid can generate electric charges through piezoelectricity, thereby promoting tissue regeneration [144]. However, the relatively weak piezoelectricity of these nanofibers limits their responsiveness to the subtle mechanical stimuli [145].

A common strategy to enhance their piezoelectric properties involves doping with specific nanoparticles to increase the crystallinity of the piezoelectric phase. MOFs are among the nanoparticles commonly used to enhance the piezoelectric response of nanofibers, particularly in sensor fabrication [146]. For instance, the incorporation of zinc-based MOFs into polyvinylidene fluoride significantly promoted the formation of the electroactive polar β-phase, with reports indicating a 20% increase in β-phase content. Such structural modifications translate into superior piezoelectric performance; polyvinylidene fluoride nanofibers doped with 1 wt% MOF crystals achieved a peak-to-peak voltage of 3.84 V under a 2.5 N applied force—a 32% improvement over pristine polymer [147]. Similarly, the integration of ZIF-8 into polyvinylidene fluoride has been shown to yield bifunctional antibacterial dressings, where 5% ZIF-8 loading resulted in a short-circuit voltage of 2.97 V and an open-circuit current of 13.7 nA. In vitro and in vivo evaluations suggest that these composites can generate reactive oxygen species under ultrasonic stimulation, where the concomitant electrical signals provide a synergistic antimicrobial efficacy [148].

However, the translation of these piezoelectric scaffolds to clinical wound care requires rigorous investigations into the stability of the MOF structure within the nanofiber matrix when exposed to wound exudates. The potential degradation or chemical transformation of the MOF in such aqueous environments may significantly alter the piezoelectric constant over time. Addressing these stability challenges could establish a new paradigm for MOF/nanofiber composites, evolving them from passive drug delivery vehicles into ‘active’ bio-electric interfaces that accelerate tissue regeneration.

## Conclusion

In summary, the synergy between Metal-Organic Frameworks and electrospun nanofibers represents a paradigm shift in advanced wound care, bridging the gap between high-capacity drug loading and precisely controlled therapeutic delivery. The diverse array of systems reviewed—ranging from pro-angiogenic HKUST-1 frameworks to highly stable UiO-66-NH₂ architectures—demonstrates the potential to achieve a near-complete healing and total eradication of resistant pathogens. However, as highlighted throughout this review, transitioning from the reported laboratory success to clinical adoption requires overcoming significant multi-dimensional challenges. Technical bottlenecks such as particle aggregation and needle clogging necessitate the adoption of more sophisticated fabrication techniques, including syringeless electrospinning and in situ crystallization.

Future research must move beyond ‘idealized’ reporting to prioritize the critical evaluation of long-term metabolic safety, environmental stability, and the integration of predictive computational tools like Machine Learning to account for the dynamic complexity of human wounds. By addressing these critical gaps—particularly the trade-off between framework stability and biological activity—MOF-nanofiber composites can evolve from experimental scaffolds into sophisticated, bio-active interfaces capable of managing the most complex chronic wound environments.

## Data Availability

Data will be available upon reasonable request from the corresponding author.
